# Micro/Nanorobot: A Promising Targeted Drug Delivery System

**DOI:** 10.3390/pharmaceutics12070665

**Published:** 2020-07-15

**Authors:** Mengyi Hu, Xuemei Ge, Xuan Chen, Wenwei Mao, Xiuping Qian, Wei-En Yuan

**Affiliations:** 1 Engineering Research Center of Cell & Therapeutic Antibody, Ministry of Education, School of Pharmacy, Shanghai Jiao Tong University, Shanghai 200240, China; humyi018@sjtu.edu.cn (M.H.); belieforever97@sjtu.edu.cn (X.C.); 2 Department of Food Science and Technology, College of Light Industry Science and Engineering, Nanjing Forestry University, Nanjing 210037, China; gexuemei@njfu.edu.cn

**Keywords:** micro/nanorobot, targeted drug delivery, exogenous power, endogenous power, cell-based micro/nanorobot, DNA origami

## Abstract

Micro/nanorobot, as a research field, has attracted interest in recent years. It has great potential in medical treatment, as it can be applied in targeted drug delivery, surgical operation, disease diagnosis, etc. Differently from traditional drug delivery, which relies on blood circulation to reach the target, the designed micro/nanorobots can move autonomously, which makes it possible to deliver drugs to the hard-to-reach areas. Micro/nanorobots were driven by exogenous power (magnetic fields, light energy, acoustic fields, electric fields, etc.) or endogenous power (chemical reaction energy). Cell-based micro/nanorobots and DNA origami without autonomous movement ability were also introduced in this article. Although micro/nanorobots have excellent prospects, the current research is mainly based on *in vitro* experiments; *in vivo* research is still in its infancy. Further biological experiments are required to verify *in vivo* drug delivery effects of micro/nanorobots. This paper mainly discusses the research status, challenges, and future development of micro/nanorobots.

## 1. Introduction

Accurate drug delivery technology has always been a hard nut to crack in the pharmaceutical research field. The most ideal condition is that the therapeutic dose of a drug is transported directly to the target organs/tissues/cells to produce a marked effect, but this cannot be achieved through traditional administration methods. The nano carrier-based drug delivery system is a promising strategy to solve this problem. The application of nanotechnology can improve the drug solubility, change the drug distribution in various tissues and organs, adjust the release rate to obtain sustained release and controlled release profiles, and promote the drug aggregation in its target [1,2]. Nanodrug delivery systems are extensively studied due to the above-mentioned strengths. Even though the scientific reports and researches on nanodrugs are massive, only a few actually enter clinical trials, and an even smaller handful can be approved [3]. It is widely-known that with passive-targeted or active-targeted nano carriers, the realization of drug delivery depends primarily on blood circulation to achieve the systemic distribution [4]. The specific physiological structure of tumor tissue is benefit for the aggregation of specific size molecules, so that passive targeting nanodrugs can be enriched to the tumor site through the enhanced permeability and retention (EPR) effect [5]. In the complex organism environment, the EPR effect is easily affected by the clearance effect of the mononuclear phagocyte system (MPS), glomerular filtration in the kidney, and the resistance of capillary wall [6]. Thus, in terms of targeting effect, the increment of nanoparticles aggregation by EPR effect in tumor tissues was less than twice as much as that in normal tissues [7]. What is more, only less than one percent of nanoparticles could accumulate in tumors [8]. As for active targeted nanoparticles, they were linked with numerous targeting ligands designed according to the gene expression difference between target cells and normal cells. These ligands include antibodies (epidermal growth factor receptor (EGFR), etc. [9]), antibody fragments, peptides (transferrin, etc. [10]), small molecules (folic acid, etc. [11]), and other different receptor ligands [2]. Although the connection between ligands and receptors can facilitate the active targeted nanoparticles’ binding effect to the target site, the carriers are actually incapable of directly finding the targets [12]. The active targeted nanoparticles are unable to discover the location of receptors in tumor tissues and approach them. Only when the nanoparticles are distributed in the target tissues could make it possible to combine the modified target head with the receptor to play a further role. This kind of active target is not a complete target; the enrichment at the target site is purely based on blood circulation through the target area [13]. Some studies proposed that the promotion of the active targeted nanoparticle’s targeting efficiency at the tumor site was not totally ideal, for example, folate receptor modified liposomes did not show a significant increase in tumor accumulation [14], and the modification of the antibody (anti-HER2) on long-circulating lipidic nanoparticles had no dramatic promotion in the accumulation at the tumor tissue [15]. In order to reach the effective drug accumulation in the target area, the measures of prolonging the circulation time of nanoparticles and increasing the dosage are usually taken, while the risk will increase at the meantime [12]. A drug delivery system is desired to possess some specific abilities to achieve accurate delivery of therapeutic payloads, including autonomous propulsion, controlled navigation, tissue penetration, payloads towing, and releasing [16]. However, these remain huge challenges for current nano carrier drug delivery systems, yet it is rather difficult to realize the true targeted delivery of drugs.

The fast development of materials science, molecular biology, mechanical dynamics, artificial intelligence, and other fields hastens the birth of new industries. Micro/nanorobot gradually entered the scientists’ horizons. In 1959, the idea of applying microrobots to medical treatment was first proposed by Richard Feynman [17]. The concept of nanorobot then came into being. As a burgeoning technology, micro/nanorobot has been widely used in medical field, its functions include auxiliary operation, medical diagnosis, and drug delivery [18]. As for drug delivery, differently from the traditional mode which relies on blood circulation to reach the target, micro/nanorobots can achieve autonomous movement, which allows us to send controlled nanoparticles to the difficult-to-access areas. Micro/nanorobot system is usually formed by an internal payload and an external shell that can reach a specific target actively [19]. The propulsion of micro/nanorobot is guided by exogenous dynamics (such as magnetic propulsion, ultrasound propulsion, and light energy propulsion [20]) or endogenous dynamics (achieved by chemical or biological reactions [21]). The existing drug delivery system depends on systemic circulation and lacks the navigation ability required for accurate delivery [16]. Micro/nanorobots with motion ability can meet these features to a certain extent and represent an attractive prospect. In this review, we present the research status of micro/nanorobots in the targeted drug delivery field according to their propulsion mode. In the meantime, the study also involves the existing challenges micro/nanorobots research encountered and its future development direction.

## 2. Micro/Nanorobots with Autonomous Movement Ability

### 2.1. Exogenous Power Driven Micro/Nanorobots

Since the size of drug delivery robot system is only micro or nano scale, it needs to overcome Brownian motion to realize its autonomous locomotion in complex body fluids. For the purpose of driving the movement of particles, an exogenous power is usually attached to control and coordinate the micro/nanorobot’s behaviors. Magnetic field [22], electric field [23], light energy [24], acoustic wave [25], and heat energy [26] are frequently applied in drug carriers as an external power. In the actual design process, several driving modes are often used jointly to manufacture micro/nanorobots with multiple functions.

#### 2.1.1. Magnetic Field Propelled Micro/Nanorobots

**Figure 1:**
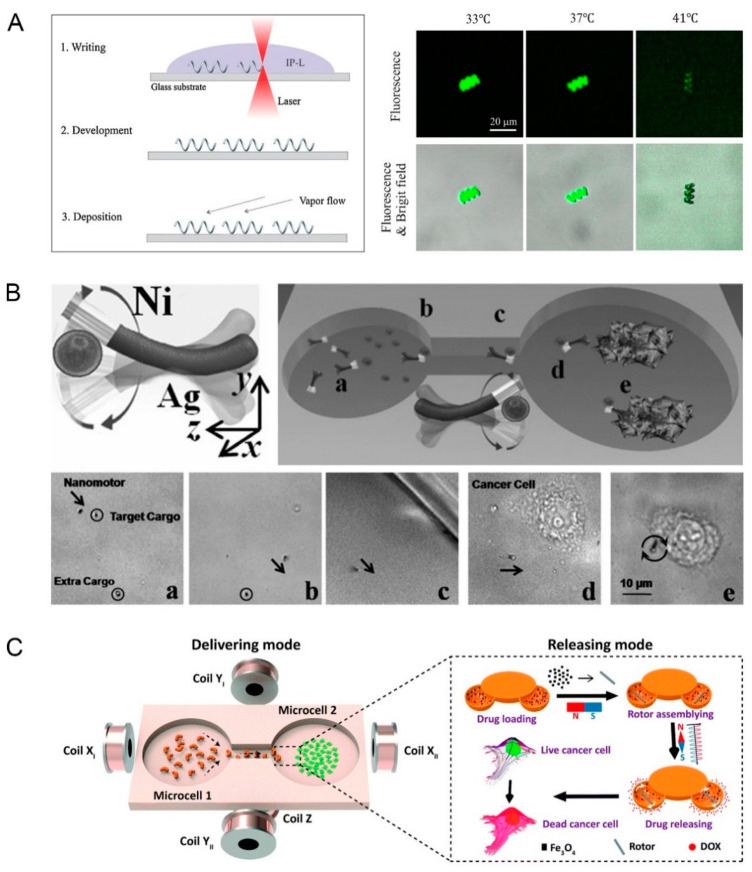
Propulsion mechanism and drug delivery of magnetic field propelled micro/nanorobots. (**A**) Helically-shaped microrobot with artificial bacterial flagella and drug-loaded liposomes (reprinted from [28] with permission from Elsevier, 2014); (**B**) Magnetic Ni-Ag nanorobot with flexible structure (reprinted from [35] with permission from John Wiley and Sons, 2012); (**C**) Pine pollen-based microrobot with Fe_3_O_4_ magnetic particles and drugs (reprinted from [40] with permission from Royal Society of Chemistry, 2019).

Among these energies, the application of magnetic fields for propulsion is the most popular. As a promising option, it can realize a variety of swimming strategies. Magnetic propulsion is usually designed as a helical swimmer, flexible swimmer, or surface walker [22]. Micro-organisms have developed special structures with helically-shaped appendages or flagella after billions of years of evolution. The design of helical swimmers is to imitate bacterial flagella’s rotary corkscrew motion. Magnetic helically shaped micro or nanorobots could be propelled and realize rotational–translational motion via a torque generated by the interaction external magnetic fields and helical shape [27]. The combination of artificial bacterial flagella (ABF) and drug-loaded liposomes is often used. Qiu et al. designed a microrobot model including two parts: Titanium-coated ABF under rotating magnetic fields could achieve precise 3D-navigation in fluids, the outermost temperature-sensitive liposome could release the drug under the temperature regulation (Figure 1A) [28]. Calcein (model drug) was successfully released about 73 ± 15% at 41 °C, which confirmed its potential application in the targeted drug delivery. The helical nanostructure could translate under the rotating magnetic fields, and its motion direction depended on the sense of rotating magnetic fields and the handedness of the helix. Qiu et al. also constructed ABFs functionalized with lipoplexes containing pDNA, which achieved successful targeted gene delivery *in vitro* to human embryonic kidney (HEK 293) cells; the magnetic Ni layer endowed the microrobot with the ability to move under low magnetic fields, but when used *in vivo*, the chronic effects caused by the long-term existence of metals requires careful consideration [29]. Furthermore, to deal with the more complex environment inside the body, more types of ABF-structure motors have been designed. Helical magnetic nanomotor was demonstrated to move and manipulate inside biological cells [30]. According to bacterial structure, an environmental responsiveness nanorobot with ABF was designed; its flagellar helical form would change in response to environmental stimuli, resulting in a difference in propulsion [31]. The research of these nanomotors contributes to the application of drug loading. Micro/nanorobots with ABF structure could realize precise movement in low magnetic field intensity (1000 times weaker than magnetic resonance imaging (MRI)), which is harmless to the human body [28]. Nevertheless, the photoresist used in ABF preparation and the metal coatings that may be added to the surface limited the further application *in vivo*.

As for the flexible swimmer, differently from other fixed robots, it has flexible tails or joints, or a bendable body [32]. Its movement is based on an undulatory locomotion mechanism, leading to a travelling wave along its flexible body. A flexible magnetic nanorobot is usually a rod-shaped structure with segment splicing, and the flexible joint in the middle is often made of silver. The simplest structure is flexible Au–Ag–Ni nanowire [33]. The Au and Ni segments would rotate at different amplitudes under the rotating magnetic field, hence breaking the symmetry of the system and triggering its motion. Jiang et al. fabricated 1-, 2-, and 3-link magnetic nanoswimmers and studied their movements under the magnetic field [32]. The results revealed that undulation motion was observed in 1- and 2-link nanoswimmers, S-like motion appeared in 3-link nanoswimmers. A nanorobot containing two gold segments (head and caudal fin), two nickel segments (body) was designed to imitate the movement of fish [34]. Three flexible porous silver hinges were added to link each segment and the fish-liked nanorobot could generate travelling-wave motions under oscillating magnetic fields. Based on these flexible structures, active drug delivery can be realized by combining drug carriers. Gao et al. firstly designed a flexible nanorobot for the direct delivery of drug-loaded particles (Figure 1B) [35]. The drug-loaded microspheres made by poly (lactic-co-glycolic acid) (PLGA) was connected to the nickel segment with strong magnetic properties of magnetic Ni–Ag nanorobot, which could be successfully delivered to HeLa cancer cells *in vitro*; and the nonspecifically bound of Ag segment to the HeLa cells allowed Doxorubicin (Dox) to release from microspheres. In such a structural design, the size ratio of drug-loaded particles to the nanomotor will affect its move ability. Gutman et al. conducted a research on the optimal size ratio between the cargo and the flexible microswimmer’s tail [36]. Although the micro/nanorobot is flexible on the whole, it is basically made of solid hard metal, each segment is still rigid. The drug loaded particles can only adhere to the metal surface, which limits the delivery effect to a certain extent. Thus, worm-like magnetic nanorobots with great potential in controlled delivery and release of cargo in biological fluids were designed [37]. The CoFe_2_O_4_ nanoparticles on the surface of worm-like mesoporous silica nanotube (MSN) could propel the movement of the nanorobot under external magnetic field. The G-quadruplex layer covered on the MSN functioned as a barrier, which was less stable under external stimuli and thus releasing nanoparticles. This system achieved the delivery of 6-carboxyfluoresceins to HeLa cells.

Surface magnetic walkers could move near surface under magnetic fields. Rotating Ni nanowires [38] and colloidal microwheels [39] were designed as surface magnetic walkers with movement ability and showed great potential in targeted drug delivery. In addition, many other types of magnetic field driven nanorobots are gradually emerging. Sun et al. designed a pine pollen-based micromotor with two hollow air sacs encapsulating Fe_3_O_4_ magnetic particles and drugs to conduct drug delivery (Figure 1C) [40]. Fe_3_O_4_ particles endowed the microrobot controllable navigated locomotion capability and 3 motion modes (rolling + tumbling + spinning); and model drug (Dox) was transported to Hela cells through the change of rotating magnetic field.

In conclusion, magnetic energy driven micro/nanorobot had the advantages of precise positioning, controllable direction, and wide range of motion. The micro/nanorobots can work under a relative weak magnetic field, which is safe and harmless. But the metal added to the core material or surface coating will limit its application *in vivo*. For example, metal trapped *in vivo* will bring immune reaction, inflammatory reaction, or other potential side effects to the human body [41,42], thus more safe and non-toxic alternative materials are needed.

#### 2.1.2. Electric Field Propelled Micro/Nanorobots

**Figure 2:**
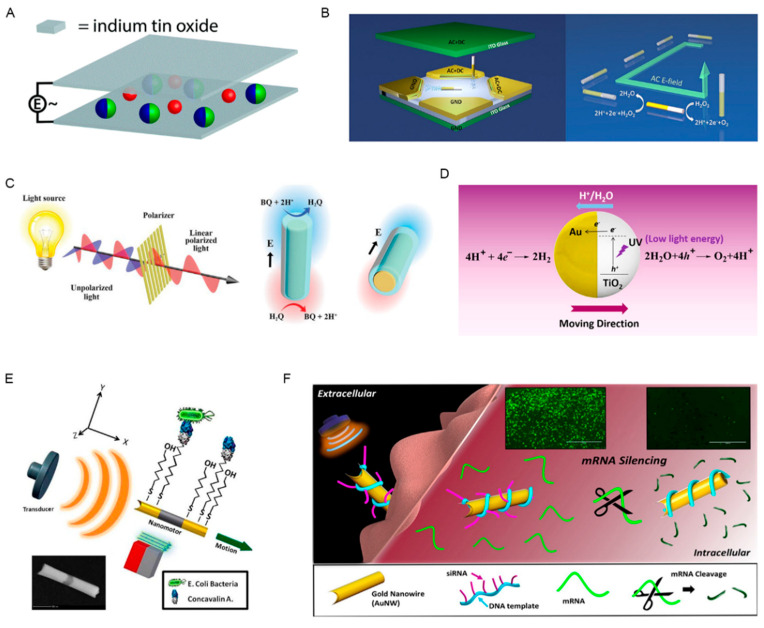
The working models of micro/nanorobots driven by exogenous power. (**A**) (reprinted from with [43] permission from Royal Society of Chemistry, 2018), (**B**) (reprinted from [23] with permission from American Chemical Society, 2018): Electric field propelled micro/nanorobot. (**C**) (reprinted from [46] with permission from John Wiley and Sons, 2019), (**D**) (reprinted from [50] with permission from American Chemical Society, 2016): Light energy propelled micro/nanorobot. (**E**) (reprinted from [55] with permission from American Chemical Society, 2013), (**F**) (reprinted from [57] with permission from American Chemical Society, 2016): Ultrasound energy propelled micro/nanorobot.

Electric energy and magnetic energy are usually inseparable and can be mutually transformed under certain circumstances. The electric field driven micro/nanorobot is also very common. A Janus colloidal system driven by electric and magnetic energy has been reported (Figure 2A) [43]. Autonomous movement and cargo pick-up ability of the metal dielectric Janus colloidal system are achieved by an external high frequency electric field (0.5–2.5 MHz). The hemisphere of the Janus colloid was coated with a nickel layer; thus, its motion direction can be steered by magnetic field. This made precise cargo delivery possible by programming the path the micro/nanorobot follows. A rotational nanomotor structure (carbon nanotube) designed by Rahman et al. displayed fast responsiveness and ultra-high speed movement under electric field [44]. The motion was originated from the electric-induced water dipole orientation, while it only showed good characteristics in water, and its effects in simulating body fluid or more complex human system were unknown. Nanoparticles can also complete directional movement under the joint effects of electric field and light energy [45]. Guo et al. reported a precise and controllable approach in regulating the catalytic nanomotor’s movement by applying electric fields (Figure 2B) [23]. Pt-based bimetallic nanorod motor, a model system, successfully realized movement in different speeds and directions through combined alternating current (AC) and direct current (DC) electric fields in three-dimensional (3D) direction and the current change. Au nanoporous templates fabricated by electrodeposition, serving as cargos, could be accurately picked up and released through electric field regulation. In the above-mentioned studies, researchers have confirmed the excellent capability of micro/nanorobots to extract, transport, release cargos, and direct movement *in vitro*, which lays a solid foundation for the drug delivery application *in vivo*. Electric energy is readily available, but its penetration is not as strong as magnetic field, which leads to the increase of the electric field intensity in actual use. The damages caused by excessive current to the human body in practical application should be taken into consideration. What is more, similar to magnetic field-driven micro/nanorobots, metals are always inevitably used in electric field-propelled micro/nanorobots manufacturing, which needs to be carefully investigated.

#### 2.1.3. Light Energy Propelled Micro/Nanorobots

Light energy is another common method and has demonstrated great controllability and programmability, and it is mainly used as an auxiliary means. Directional movement of the nanorobot is realized by modulating the light frequency, polarization, intensity, and propagation direction [24]. Due to the nanowire linear dichroism of Sb_2_Se_3_, Zhan et al. designed an artificial swimmer by integrating two cross-aligned dichroic nanomotors; its movement can be navigated by regulating the incident light’s polarization direction (Figure 2C) [46]. Although the authors did not carry out follow-up experiments, this model might be effective for targeted delivery of drugs. Apart from the function mode of directly driving the movement of micro/nanorobot, light energy can also play a catalytic role in inducing the redox reaction inside the micro/nanorobot and further propel the nanorobot by producing chemical gradients or bubbles [47,48]. A glucose-fueled Cu_2_O@N-doped carbon nanotube (Cu_2_O@N-CNT) micromotor was designed by Wang et al., which could be activated by visible-light photocatalysis [49]. It was non-toxic, highly biocompatible, environmentally friendly, and could realize outstanding movement and 3D motion control in biological environment. Although *in vitro* studies have shown excellent results, its application in biological system remains a challenge due to the inability of visible light to penetrate tissues. Photocatalytic TiO_2_–Au Janus micromotor triggered by ultraviolet (UV) light showed good performance in driving ability (Figure 2D) [50], while exposure to UV light will cause harmful effects and thus limit its application. Near infrared (NIR) light can penetrate tissues with minimal side effects and has drawn widespread attention recently [51,52]. Xuan et al. demonstrated a nanorobot system which could achieve a directional motion when exposed to NIR light (the Au half-nanoshell could generate a heat gradient and form a self-thermophoretic energy to overcome the Brownian motion) [53]. This system was also coated by macrophage cell membrane, endowing the nanorobot with immunological properties to actively recognize and bind cancer cells. Although light energy driven by nanorobot is promising, most existed researches are conducted *in vitro*. When facing complex internal environment, it is worth deeply exploring whether its directional movement can perform as good as *in vitro*. Additionally, micro/nanorobots driven directly by light energy for drug delivery are rare: Light energy will work with other energy to propel the micro/nanorobots’ movements in most cases, or trigger the release of drugs at the target site.

#### 2.1.4. Ultrasound Energy Propelled Micro/Nanorobots

Due to the excellent biocompatibility and reliability, ultrasound-powered nanorobots present great potential in active targeted drug delivery. Nanowires are the common carrier of ultrasonic driven nanorobots, which are usually made of gold [25]. The template electrodeposition method is heavily used in the design process of ultrasound-propelled micro/nanomotors: Firstly, a concave cavity was created by the copper sacrificial layer deposition in one end of the nanomotor; when the ultrasound waves penetrating the concave end, the nanomotor could move under the propulsion of the generated pressure gradient [54]. Ultrasound is commonly combined with magnetic field. Victor et al. designed a magnetically guided three-segment Au–Ni–Au nanowire motor which was propelled by ultrasound (Figure 2E) [55]. The change of the applied magnetic field direction induced omni-directional movement of ultrasound-propelled particles. The capability of targeted drug delivery has been confirmed through adding a polymeric (PPy-PSS) segment loaded with pH sensitive drugs into the nanomotor, the drug in which could release in acid environment. An ultrasound-propelled nanorobot with four segments including Au–Ni–Au and Au wire was produced by Victor et al. [56]. Nanoparticle’s movement towards cancer cells was driven by ultrasound, and loaded drug release was triggered by NIR light stimulation. After loading Dox into this model, it was travelled towards the HeLa cell, and 38% of the drug was released after 15 min of NIR irradiation. A gold nanowire wrapped with a rolling circle amplification DNA strand that could hybridize with siRNA was designed for intracellular siRNA delivery (Figure 2F) [57]. Its directional motion was induced by the pressure gradient generated by ultrasound. Ultrasound provided the nanorobot with a fast and large thrust, so the nanorobot could effectively penetrate into the cell, and then siRNA acted as scissors to split the target mRNA with a 94% silencing efficiency after few minutes’ treatment. Compared with other energy driven methods, ultrasound has enough penetration and can provide strong propulsion ability for nanorobots to overcome the barriers generated in the complex environment of human body. However, the application of ultrasound might cause oxidative stress in cells [58], which might affect normal cells besides the targeted cells.

### 2.2. Endogenous Power Driven Micro/Nanorobots

**Figure 3:**
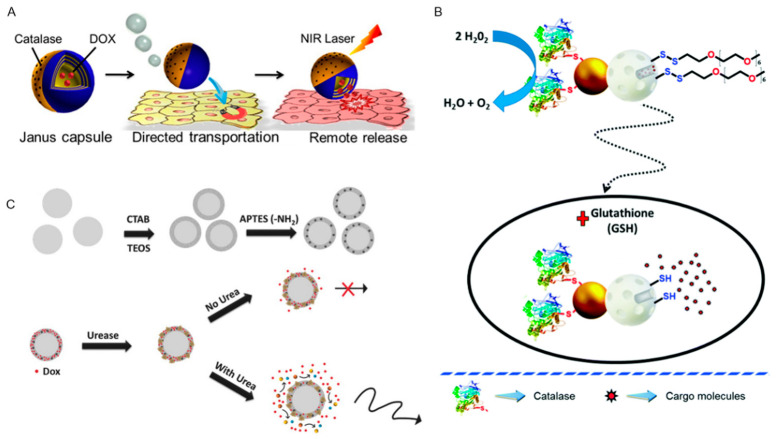
Self-propelling nanorobots by transforming chemical reaction energy into motion force. (**A**) (reprinted from [61] with permission from American Chemical Society, 2014), (**B**) (reprinted from [64] with permission from ROYAL SOCIETY OF CHEMISTRY, 2019): Hydrogen peroxide decomposition reaction provided motion energy for nanorobot. (**C**) Decomposition of urea into ammonia and carbon dioxide provided motion energy for nanorobot (reprinted from [68] with permission from John Wiley and Sons, 2018).

The endogenous power promoting the self-propelling of nanorobots is mainly produced by chemical reaction or biological reaction [59]. This type of micro/nanorobot is always asymmetric and often coated with catalyzers to obtain continuous chemical energy from the environment. Conversion of chemical energy into a driving force on a nanorobot based on redox reaction is a quite popular scheme. Particularly, a decomposition reaction of hydrogen peroxide was the most frequently used method. Hydrogen peroxide has unstable chemical bond and can be easily decomposed into water and oxygen under the action of various catalysts, such as metals, enzymes, and an alkaline environment. Furthermore, it was widely applicated in nanorobots in the forms of bimetallic nanorods [60], hollow Janus particles [61], vesicular polymer [62], etc. This kind of nanorobot has shown promising prospects in targeted drug delivery field [63,64]. Janus particles are nano- or micron particles with different composition and structure in two hemispheres. Wu et al. fabricated a polymer multilayer Janus capsules, which was self-propelled in 0.1% hydrogen peroxide fuel at physiological temperature [61]. As shown in Figure 3A, the core of the Janus capsules was SiO_2_ particle covered with 5 layers of PSS/PAH; Ni and Au were only covered on the surface of one hemisphere. The catalase on the Au layer could catalyze the breakdown of hydrogen peroxide, and the resulted gas drove the motion of the system. Under the guidance of magnetic field, the particles moved to the target. Then, the outer polymer shell was destroyed under NIR, thus the drug carried was released to work. The materials used for nanorobot manufacture are all biocompatibility or biodegradability, such as polymer materials, gold, and enzymes. Although it has been confirmed that almost all HeLa cells could survive for more than 3 h in 0.2% H_2_O_2_ environment *in vitro* experiment, the toxicity of H_2_O_2_ fuel itself could not be ignored. Antoni et al. reported a stimulus-responsive nanorobot system based on Janus Au–mesoporous silica nanoparticle with the ability of self-propelling produced by hydrogen peroxide decomposition reaction (Figure 3B) [64]. In order to identify environmental information and release payload, the disulfide-linked oligo (ethylene glycol) (SS-OEG) chains were connected to the surface, then disulfide bonds would break and release drugs when exposed to glutathione. Although this chemical energy conversion method has been studied frequently, the deep exploration of this nanorobot in organism has been greatly limited due to the toxicity of “fuel” hydrogen peroxide. Magnesium with high biocompatibility could react with water and form bubbles as impetus, which was an excellent substitute material to replace the toxic hydrogen peroxide [65,66]. Biocompatible enzyme catalysis reaction was another alternative approach to generate self-propulsion [67]. Nontoxic fuels such as glucose and urea were used to drive nanorobots. AnaC et al. reported a mesoporous silica-based core–shell nanorobot with self-propelling ability in ionic media (Figure 3C) [68]. Urease was used to functionalize the nanorobot as a catalyst to stimulate the decomposition of urea into carbon dioxide and ammonia, so that the nanorobot was able to move autonomously and release drugs. The above nanorobots loaded with Dox demonstrated high efficiency toward HeLa cells, which was related to the synergistic effect of the enhanced drug release and the ammonia from catalytic reaction.

The advantage of endogenous chemical energy drive is that it does not need to control the micro/nanorobots all the time, but needs to guide the final target position, such as using the attraction of magnetic field. The way that the gas generated by the chemical reaction reverses the robot’s movement, which is suitable for the gastrointestinal tract environment. However, some defects exist in chemical energy driven self-propelled nanorobot, for example, its movement direction is difficult to control, and its movement is easily disturbed by the ionic medium. The continuity of movement is one of its limitations, the micro/nanorobots may lack power in the later stage with the development of the chemical reaction. Another substantial challenge towards its application in organism is the safety of “fuel” and reaction products. These problems deserve further exploration in the future. See Table 1 for more information about the characteristics of endogenous power-driven micro/nanorobot and its comparison with exogenous power-driven micro/nanorobot.

## 3. Other Types of Micro/Nanorobots

**Figure 4:**
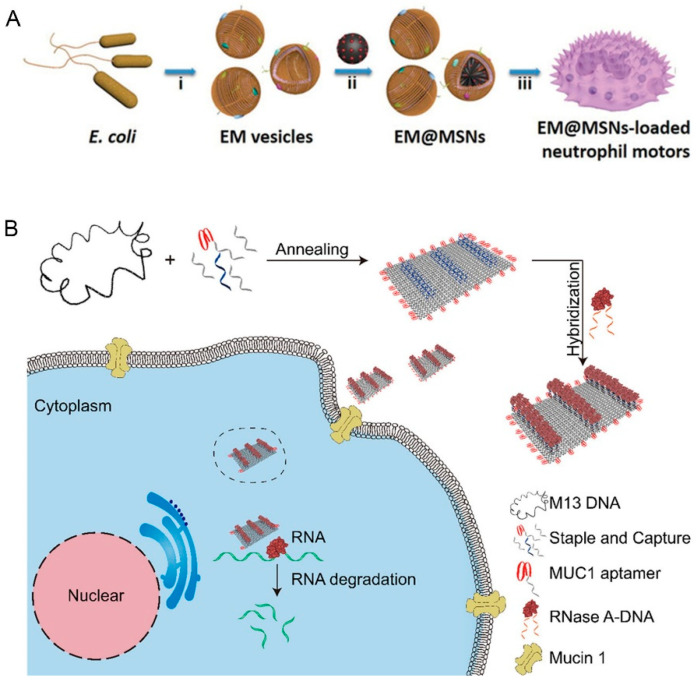
Other nanorobots with the enhanced targeted drug delivery ability. (**A**) Chemotaxis-guided hybrid neutrophil micromotor used for targeted drug delivery by (reprinted from [69] with permission from John Wiley and Sons, 2017). (**B**) DNA origami nanorobot used for RNase A delivery (reprinted from [78] with permission from American Chemical Society, 2019).

Cell-based micro/nanorobots systems such as red blood cells, bacteria, and stem cells are considered to be effective and biocompatible for targeted drug delivery [65]. The excellent escape mechanism and biocompatibility of cells and microorganisms can be transmitted to micro/nanorobots, which assist the targeted drug delivery. Nevertheless, the coating of the cell membrane can only reduce the recognition of the environment, and cannot realize the directional movement, which still needs the stimulation of external magnetic field, sound wave, etc. Shao et al. designed self-guided hybrid micromotors consisting of neutrophils with chemotaxis ability and mesoporous silica nanoparticles (MSNs) with loading ability (Figure 4A) [69]. As *E. coli* were coated on MSNs loaded with Dox in advance, the designed system could move effectively along the chemoattractant gradients produced by *E. coli*. The magnetic nanoparticles (MNPs) within the red blood cells (RBC) allow for efficient magnetic guidance and movement under ultrasound propulsion [70]. The RBC microrobot loaded with Dox, quantum dots (QDs) imaging agents and MNPs could move and transport drugs along established routes. In addition, its cytotoxicity was three times lower than Dox and QDs alone. Magneto-aerotactic bacteria is also commonly used, Ouajdi et al. reported that the *Magnetococcus marinus* strain MC-1 combined with drug-containing nanoliposomes showed more than 55% cells penetrating into the targeted tumor hypoxic regions to achieve the drug delivery [71]. As the objects nanorobots can imitate in the future, the movement characteristics and modes of multifarious microorganisms and cells *in vivo* are worthy of exploration.

DNA origami, another new technology, is commonly used to construct DNA nanorobots and has great potential to be applied in intelligent drug delivery field. Based on the classic principle of complementary base pairing, a single-stranded DNA is folded repeatedly and fixed by many short oligonucleotides used as “staple strands”, so that the designed DNA nanostructures can be obtained [72]. DNA origami has excellent addressability for functional ligands, biomolecules, or nanoscale objects to be precisely organized on the desired position of its surface, which promotes the targeting ability of DNA origami nanorobots [73]. Although DNA origami robots usually do not have the ability to move autonomously, they show great target drug delivery capability [74,75]. Li et al. designed a programming rectangular DNA origami nanorobot (20 nm × 30 nm), which effectively penetrated ovarian cancer cells when loaded with adriamycin [76]. DNA origami nanorobots were also reported to successfully deliver thrombin or ribonuclease (RNase) A molecules to the target cells [77,78]. Capture strands (blue) linked to cytotoxic protein RNase A molecules were extended on the surface of rectangular DNA origami template, and aptamers targeting cancer cells are also integrated to enhance the targeting effect (Figure 4B) [78]. RNase A molecules were successfully delivered into the cytoplasm and played a cell-killing role inside the tumor cells. However, the retention time of nanorobots *in vivo* remains a challenge because of the clearance effect of activated immune system. Methods based on DNA origami structure will be devised to realize the directional motion of nanorobots in the future.

## 4. Application of Drug-Loaded Micro/Nanorobots *In Vivo*

In the above-mentioned contents, micro/nanorobots driven by different kinds of energy have been introduced through numerous examples, their directional motion and drug delivery potential have also been well demonstrated *in vitro* studies. Application of these drug delivery systems *in vivo* will be displayed in this part along with the difficulties that need to be overcome during the transition from laboratory to clinic.

The gastrointestinal tract is relatively easier to access, thus there are more researches on gastrointestinal tract drug delivery than other in other fields. In gastric tissue, the acidic environment is usually used to achieve drug delivery, such as promoting the movement of nanorobots by hydrogen produced by the reaction of metals and hydrogen ions. Esteban-Fernández de Ávila et al. designed a Mg-based microrobot loaded with clarithromycin (CLR) to treat mice with gastric infection [79]. *H. pylori*-infected mice were orally administered with the microrobots to treat gastric infection. The results showed the CLR-loaded microrobots group reduced approximately 1.8 orders of magnitude of the *H. pylori* burden than negative control group (treated with deionized water or microrobot without CLR). In addition to the therapeutic effect, the authors demonstrated the safety and non-toxicity of this system in mice. A similar Zn-based microrobot was also produced by Gao et al. [80]. Gold nanoparticles (AuNPs), used as model drug, were successfully delivered and retained to the stomach of the mice by microrobot, at more than three times the rate of orally-administrated NPs group. The safety of microrobots was confirmed by the gastric tissue sections of mice taking the microrobots.

Wu et al. utilized photoacoustic computed tomography (PACT) technology to realize deep imaging of microrobotic system in intestines [81]. The enteric coating was covered outside the Mg-based micromotors to form a micromotor capsule. When the capsule reached the intestine, its phase transition was induced by NIR, thus the micromotors were released due to the broken of the capsule shell. The chemical reaction between Mg and water is the energy source to drive the micromotor. As the microrobots were gradually dissolved, the drugs were slowly released. Dox in the alginate layer of the micromotors was delivered successfully to the intestine of mice.

Unlike other body fluids, there are many free-moving cells and other contents in the blood, which bring more obstacles to the micro/nanorobots’ movement. Self-propelled particles based on the chemical reaction of carbonate and tranexamic acid were devised by Baylis et al. [82]. The thrombin adsorbed on porous particles was used to halt hemorrhage through blood vessel in mice and pigs. The research confirmed that this system was capable of stable movement in the blood and of delivering thrombin for hemostatic treatment. Recently, new model microrobots like sperm micromotors [83], multifunctional surface micro rollers [84] have also been designed for intravascular drug delivery.

Furthermore, there are also studies involving the delivery of drugs to tumors, eyes or other areas. The above-mentioned (Section 3) drug delivery system based on *Magnetococcus marinus* strain MC-1 could deliver the SN-38 drug-loaded liposomes into hypoxic regions of HCT116 colorectal xenografts in mice [71]. Kim et al. produced a bilayer hydrogel microrobot to transport drug particles to eyes, which could achieve target delivery and be recycled to avoid adverse reactions caused by its retention in the body [85]. The MNPs layer brings the microrobots to the treatment site, and then the PLGA–Dox drug particles were transferred to the lesion after the therapeutic layer dissolving under an alternating magnetic field. At last, using an external magnetic field could retrieve the remaining MNPs layer.

In addition to drug delivery, cell transportation *in vivo* is widely used in stem cell therapy. A 3D porous structure micro/nanorobot driven by magnetic field is an ideal strategy to precisely deliver stem cells to therapeutic sites for tissue repair. Li et al. designed microrobots with burr-like porous spherical structure, which were able to deliver the HeLa cells to nude mice *in vivo* [86]. MC3T3-E1 fibroblasts and mesenchymal stem cells (MSCs) cultured on culture plates with the same coating as the microrobot (Ni and Ti) for 1, 3, and 5 days confirmed the safety of the microrobots. This 3D microrobot was biocompatible but not biodegradable. Jeon et al. also designed a porous 3D microrobot with similar structure [87]. Under the function of magnetic field, the microrobots loaded with MSCs delivered cells in the intraperitoneal cavity of a nude mouse. However, in such a transport process, cell viability may be affected by fluid shear force and magnetic field. Cells loaded micro/nanorobot was also applied in knee cartilage regeneration *in vivo* [88]. The human adipose–derived MSCs (hADMSCs)-located microrobot could move to the lesion of rabbit knee cartilage under the guidance of magnetic field. Microrobots would degrade in 3 weeks without causing any inflammation in rabbits and the hADMSCs were implanted on the lesion site. The applications of drug-loaded micro/nanorobots in animal models in recent years were summarized in Table 2.

Reasonable design of directional motion and cargo delivery function in micro/nanorobot is only the first step, micro/nanorobots must clear some major hurdles before clinical researches: (1) Safety: The materials used for micro/nanorobots must be biocompatible (polymers; proteins; DNA etc.) and biodegradable (gelatin, alginate, mesoporous silica etc.) [89]. From these *in vivo* studies described above, we can discover that most materials used in micro/nanorobots guarantee biocompatibility, but less degradability. (2) Motion performance in complex body fluids: The liquid environment for *in vitro* experiments is mostly based on Newtonian fluids; however, the body fluid environment is much more complex [90]. Micro/nanorobots perform well *in vitro* while their accurate positioning and directional motion ability *in vivo* remained uncertain. When facing vessels with complex structure and different thickness, body fluids with different composition and viscosity, nanorobots need stronger capability to move freely and realize directional transportation. Brownian motion and aggregation behavior are also need to be overcome when the robot is reduced to nanometer and micron size (3) Manipulation and positioning of micro/nanorobots after entering deep tissues: Drugs usually need to be delivered to deep organs, tissues, and cells, so only with an excellent ability to manipulate and locate can micro/nanorobots be better guided to target sites. In order to deal with the complex environment *in vivo*, the operation of micro/nanorobots in deep tissue needs the guidance of imaging technology. (4) Micro/nanorobots’ fate after the accomplishment of tasks: An ideal micro/nanorobot system is expected to be safely cleared by the body upon completion of the tasks. If micro/nanorobots were retained in the body after mission completion, they might accumulate in organs and cause chronic inflammatory reactions or other adverse effects. The solutions are broadly divided into three types: Manufacturing micro/nanorobots with safe and biodegradable materials; recycling micro/nanorobots through some technical methods; triggering their self-degradation through external stimuli [91]. Although some solutions have been issued, only preliminary safety validation has been carried out, and more investigations are in need to verify their application *in vivo*. 

## 5. Summary and Prospect

In this review, the recent research status of targeted drug delivery micro/nanorobots was summarized. Due to the requirement of precise targeted drug delivery, micro/nanorobots with an exogenous or endogenous power to promote the directional movement were designed. The exogenous power-propelled micro/nanorobots can be driven by magnetic fields, light energy, acoustic fields, electric fields, etc. It has precise positioning control ability, but is easily disturbed by internal environment, and needs special and complex operation steps. The self-propelled micro/nanorobot is usually supported by chemical reaction energy, which has the virtues of low cost and convenient operation. But its motion direction cannot be controlled properly; the power might be insufficient and hard to the drive micro/nanorobot in the later stage with the reaction going on. The safety and biocompatibility of fuel and reaction products should also be taken into careful consideration. In addition to directly controlling micro/nanorobot’s movement, other approaches to promote the targeting ability are also introduced. Cell-based micro/nanorobots systems have great biocompatibility and can realize immune escape *in vivo*, so that the targeting capability is enhanced. As a new technology, DNA origami also performs well in targeted drug delivery.

Although the number of scientific researches on micro/nanorobots shows a state of rapid growth, it is still in its infancy in practical application. The convert process from theory to practice faces great challenges. In terms of mechanical structure, it is relatively hard to operate when the robot is reduced to nanometer size. In addition, no battery is small enough to power a micro/nanorobot [19]. The target delivery of micro/nanorobots in physiological environment needs to overcome more obstacles. Firstly, the smaller the particle is, the more obvious the irregular Brownian motion is; in order to realize directional motion, micro/nanorobot must overcome Brownian motion. Furthermore, when robots are downsized to nano scale, their aggregation behavior in liquids should also be taken into account, which will disturb the micro/nanorobot’s directional movement [92,93]. Among the published researches, the majority of the studies about nanorobots were only reported the *in vitro* experiments with good performance. But when it comes to complex body fluid environment, it is doubtful whether the targeted drug delivery ability of nanorobots can be maintained. Good models *in vitro* are not necessarily suitable for practical treatment. For example, visible light-driven nanorobots are unable to be applied to human body because visible light cannot penetrate tissues. As for *in vivo* studies, the safety of the micro/nanorobot was only verified in a short period of time (several days or weeks) in most researches, and the long-term hidden dangers of micro/nanorobots *in vivo* have not been fully investigated. More in-depth researches are still needed on suitable materials for micro/nanorobots to avoid the adverse effects caused by the staying of micro/nanorobots in the body for a long time. Meanwhile, it is essential to turn from *in vitro* experiment to biological experiment so as to further verify the applicability of the designed micro/nanorobot.

An ideal micro/nanorobot requires precise targeted movement and autonomous drug delivery abilities. It should also be non-toxic, can be discharged from the body or biodegraded after the action, and stable in the external environment. Other factors like real-time tracking control, visualization *in vivo* can be introduced into nanorobot as well. Bionics is also a good inspiration in the design of nanorobots, such as mimicking red blood cells that can swim freely in blood vessels.

With the development of new materials and 3D printing technology or other advanced techniques, it is believed that more precise drug delivery nanorobots will be developed.

## Figures and Tables

**Table 1:** Comparison of exogenous and endogenous power-driven micro/nanorobots.

Type	Energy	Penetration	Move Ability	Persistence	Safety
Exogenous power	Magnetic fields	Good, can work under a relative weak magnetic field	Precise 3D-navigation in fluids under rotating magnetic fields	Good, micro/nanorobots can keep moving with the guidance of external forces	The magnetic field used is within safe range; metal materials will bring potential harm to human body
	Electric energy	Relatively weak, need to increase the electric field intensity	Directional movement under the combination of electric energy and other energy		Strong electric field intensity may affect human body; metal materials will bring potential harm to human body
	Light energy	The transmittance of different light (visible light, UV, NIR, etc.) is different	Usually function as a trigger for other reactions, can achieve directional movement		Ultraviolet light is harmful, other lights are basically safe.
	Ultrasound energy	Good, with a strong penetration ability	Usually combined with magnetic field, can achieve directional movement		Ultrasound might cause oxidative stress in cells (affect normal cells); metal materials will bring potential harm to human body
Endogenous power	Chemical energy	Not applicable	With the movement ability, but still need to be positioned by external forces (such as magnetic attraction)	Not so good, chemical energy may be depleted; when the energy decreases gradually, the motion performance of the micro/nanorobots can not be guaranteed either.	The safety of fuel needs to be considered, H_2_O_2_ is noxious, glucose and urea are nontoxic fuels

**Table 2:** Application of drug-loaded micro/nanorobots in animal models in recent years.

Types of Micro/Nanorobots	Drug Delivery Method	Target Site	Safety	Ref ^1^
Mg-based core–shell composite loaded with drug and driven by chemical energy	The positively charged chitosan outer coating adhered to the stomach wall and led to the drug release	Stomach of mice	No effect on the body weight, apparent alteration of gastrointestinal tract histopathology or observable inflammation in the mice orally administered with micromotors for 5 days	[79]
Zn-based microtube loaded with gold nanoparticles and driven by chemical energy	Microrobot gradually dissolved in the gastric acid, autonomously released their carried payloads	Stomach of mice	No gastric histopathologic change and toxicity in the mice orally administrated with micromotors	[80]
Mg-based micromotors covered by an enteric coating and driven by chemical energy	The capsule shell was destroyed by NIR, and the drug was released in the process of gradual dissolution of the microrobot	Intestine of mice	Materials (Mg, Au, gelatin, alginate, enteric polymer) were biocompatible; had no toxicity to mice taking two days continuously	[81]
Self-propelled particles loaded with drug and driven by chemical energy	Thrombin played a role in the process of particles being transported throughout blood	Vessels of tail-amputated mice, mice with liver incision; vessels of pigs with carotid artery perforation	All mice remained healthy during a single-dose test for toxicity lasted for 3 days; No signs of distress, tissue necrosis or increase in the infiltration of inflammatory cells in histological sections of the tail	[82]
Magneto-aerotactic bacteria loaded with drug-containing nanoliposomes and driven by magnetic field	The drug was released from liposomes after reaching the target site	Hypoxic regions of tumor in SCID Beige mice	No inflammation, blood counts changes, abnormal biochemical parameters in mice injected with MC-1 intravenously for 6, 24, 72 h	[71]
Bilayer hydrogel microrobot loaded with drug particles and driven by chemical energy	The therapeutic layer dissolved when heated by an alternating magnetic field, delivering drug particles to the lesion	Bovine vitreous	The remaining microrobots could be retrieved using a magnetic field	[85]
Burr-like porous spherical microrobots loaded with cells and driven by magnetic field	The carried cells were released from the microrobot and attached to the tissues after reaching the target	Dorsum of a nude mouse	Cell experiment for 1, 3 and 5 days confirmed the safety of the microrobot	[86]
Porous 3D microrobots loaded with stem cells and driven by magnetic field	After reaching the target, the cells adhered to and proliferated within the tissue	Intraperitoneal cavity of a nude mouse	The microrobot was biocompatible, but its safety *in vivo* was not mentioned	[87]
Porous 3D microrobots carried with stem cells and driven by magnetic field	Cells would adhere to the tissues when reaching the target	Knee cartilage of rabbit	Microrobots would degrade in 3 weeks without causing any inflammation in rabbits	[88]

^1^ Ref: reference.
